# Collagen I Induces Preeclampsia-Like Symptoms by Suppressing Proliferation and Invasion of Trophoblasts

**DOI:** 10.3389/fendo.2021.664766

**Published:** 2021-08-06

**Authors:** Yinglin Feng, Xia Chen, Huiqiao Wang, Xueping Chen, Zixin Lan, Pan Li, Yingshi Cao, Mian Liu, Jin Lv, Yun Chen, Yu Wang, Chao Sheng, Yingying Huang, Mei Zhong, Zhijian Wang, Xiaojing Yue, Liping Huang

**Affiliations:** ^1^Department of Obstetrics and Gynecology, Nanfang Hospital, Southern Medical University, Guangzhou, China; ^2^Department of Obstetrics and Gynecology, Foshan First People’s Hospital, Foshan, China; ^3^Zhujiang Hospital, Southern Medical University, Guangzhou, China; ^4^Microbiome Research Center, University of New South Wales, Sydney, NSW, Australia; ^5^Department of Pathology, Foshan First People’s Hospital, Foshan, China

**Keywords:** fibrosis, collagen I, preeclampsia, placenta, pathogenesis

## Abstract

Preeclampsia is a common obstetric disorder affecting 2-8% of pregnancy worldwide. Fibrosis is an important histological change occurring in preeclamptic placenta, and might depend on the excess deposition of collagen I. However, the role of fibrotic placenta and collagen I in the pathogenesis of preeclampsia remains unclear. Therefore, we analyzed the collagen deposition and the expression of Collagen I in human placenta by Masson staining, Sirius red staining and western blotting. Further, the role of collagen I in preeclampsia pathogenesis was studied in C57BL/6 mice. HTR-8/SVneo cells were used to investigate the mechanisms underlying the effects of collagen I in trophoblasts by transcriptome sequencing and pharmacological agonists. Human preeclamptic placenta exhibited a significantly higher degree of fibrosis in stem villi and terminal villi than normal placenta, and was characterized by collagen I deposition. *In vivo*, a single injection of collagen I on gestational day 0.5 led to an increase in systolic pressure of pregnant mice from gestational days 4.5–17.5, to a decrease in weight and number of embryos, and to enhanced placental collagen I expression and degree of fibrosis compared with control mice. *In vitro*, collagen I attenuated the proliferation and invasion of HTR-8SV/neo cells. This effect could be reversed by treatment with agonists of ERK and β-catenin. Moreover, transcriptome sequencing demonstrated that signaling pathways related to cell proliferation and invasion were significantly downregulated in HTR-8SV/neo cells. Thus, we propose that collagen I induced preeclampsia-like symptoms by suppressing the proliferation and invasion of trophoblasts through inhibition of the ERK phosphorylation and WNT/β-catenin signaling pathways. Our findings could pave the way to the discovery of small-molecule inhibitors for preeclampsia treatment and future studies with larger sample size are required.

## Introduction

Preeclampsia is a common, pregnancy-specific disorder that threatens 2–8% of pregnancies globally and constitutes one of the leading causes of maternal and perinatal mortality worldwide (1). It is characterized by the occurrence of new-onset hypertension, accompanied by proteinuria or other end-organ dysfunction after 20 weeks of gestation in previously normotensive women (2). To date, the pathogenesis of this morbidity is poorly understood. The placenta is generally perceived as the primary organ during disease development because the removal of placenta is necessary for symptoms to regress (3). Previous research has shown that histological changes exist in pre-eclampsia (PE) placenta include chronic inflammation, vascular lesions, villous coagulation, and villous fibrosis. Among the changes occurring in these placentae, fibrosis is an important feature (4). In particular, microscopic comparison with control placenta has revealed that villous fibrosis is more frequent in preeclamptic placentas (4, 5). Notably, fibrosis in preeclamptic placenta is reported to be related to the activation of stromal fibroblasts (6). Whether placental fibrosis is involved in the pathogenesis of preeclampsia is still unclear.

Fibrosis is a well-known pathogenic process resulting in the progressive loss of organ structure and function. It is defined by overproduction of the extracellular matrix (ECM) in connective tissues and plays a significant role in the impairment of organ function, such as during liver cirrhosis and cardiac fibrosis (7, 8). It is noteworthy that diverse diseases in different organs are associated with common pathogenic pathways, as excessive ECM deposition causes physical organ deformation, which impairs organ function and ultimately induces organ failure (9).

Collagens are the predominant components of the ECM (8, 10). Consequently, increases in collagen expression and deposition are directly associated with fibrosis (11). The family of collagens comprises twenty-eight members and regulates cell proliferation and migration, cell-matrix interactions, and cell signaling (12). Furthermore, emerging studies have indicated that collagen exerts contrasting effects on the physiology of different organs. For example, collagen could stimulate angiogenesis in a myocardial infarction model (13). Moreover, collagens have been demonstrated to reduce melanoma cell proliferation and invasion by inhibiting the FAK/PI3K/AKT pathway (14).

Collagen I is well known as one of the main member of the collagen family, constituting over 90% of the collagen of the body (15, 16). Higher production of Collagen I was once detected in preeclamptic placenta (17). Takako and his colleagues indicated that the fibroblasts isolated from preeclamptic placenta showed a higher expression of collagen I (6). Although the similar studies were limited, that collagen I may be the characteristic collagen deposited in preeclamptic placenta, and may influences the placental function, warrants further investigation.

To date, detailed investigation of the collagen I in preeclamptic placenta is lacking. Whether placental fibrosis is involved in the pathogenesis of preeclampsia is still unclear. In this study, we hypothesize that exacerbation of fibrosis leads to impairment of placental function and is involved in the pathogenesis of preeclampsia. Therefore, we performed *in vivo* and *in vitro* experiments to clarify the relationship between placental fibrosis and preeclampsia.

## Materials and Methods

### Collection of Human Placenta

Women with PE and normotensive pregnant (NP) women without previous treatments were recruited in the third trimester from March 2018 to 2019 in the Department of Obstetrics of the Nanfang Hospital, Southern Medical University, China. According to the current American College of Obstetricians and Gynecologists criteria (1), preeclampsia is diagnosed as maternal systolic blood pressure blood pressure ≥140mmHg and/or diastolic blood pressure ≥90mmHg on at least two occasions, four hours apart, with proteinuria, after 20 weeks of gestation in previously normotensive women. In the absence of proteinuria, patients with new-onset of hypertension with the new onset of any of the following: thrombocytopenia, renal insufficiency, impaired liver function, or pulmonary edema can be induced. The clinical characteristics of placenta donors are summarized in **Supplementary Table S1**.

Fresh placentae were obtained immediately after delivery. The tissue was collected from the area near the umbilical cord (1 cm from the cord insert), from both the maternal and fetal side of the placenta.

A sample size of 10 placentae in each group was used for the identification of collagen and quantification of collagen I by Masson staining, Sirius red staining, and Western blotting. The experimental procedures were detailed in the **Supplementary Material**. Following staining, the placentae were assessed for the degree of fibrosis in a blinded fashion by a trained pathologist. For Masson trichrome straining, collagen fiber was stained blue, while cytoplasm and red blood cells were stained red and nucleus blue and brown. Fibrosis area% was calculated in µm digitally using the software NDP.view2 (Hamamatsu Photonics, Hamamatsu, Japan). Sirius Red staining is a method used for collagen identification. Collagen I appears red-yellow when observed under a polarizing microscope. Each analyzed field was chosen randomly and the positive red-stained areas and red-yellow density were quantified using computerized image analysis software (NIH, MD, USA).

The collection of placentae was approved by the Ethics Committee of Nanfang Hospital(NFEC-2017-055). All participants gave written consent prior to donating their placenta.

### Collagen I Preparation

Collagen I (C7774, Sigma-Aldrich, St. Louis, MO, USA) was dissolved in 0.1 mM acetic acid and emulsified in an equal volume incomplete Freund’s adjuvant (IFA) (F5506, Sigma-Aldrich, St. Louis, MO, USA) to obtain a 1000 μg/ml solution as the stock solution.

### Animals and Experimental Protocol

This project was performed in accordance with animal protocol procedures approved by the Department of Laboratory Animal Sciences, Southern Medical University (L-2019216), and the animals were handled according to the guiding principles published in the National Institutes of Health Guide for the Care of Animals.

Female C57/BL6 mice strain (8 weeks, weight 18–20 g, purchased from the Animal Laboratory Center of Southern Medical University) were used in the experiments. They were maintained on a 12 hour/12hour light/dark schedule with free access to food and water. All female mice were housed with male mice at a 2:1 ratio overnight, and pregnancy was confirmed by the presence of vaginal spermatozoa. The presence of spermatozoa was indicative of gestational day 0.5 (E0.5d).

Pregnant female mice were randomly divided into five groups on E0.5d(six mice per group): pregnant control group, 0.5 mg/kg collagen I-treated group, 5 mg/kg collagen I-treated group, IFA-treated group, and NG-nitro-L-arginine methyl ester (L-NAME)-treated group. In the collagen I-treated groups, 100 μL of collagen I (0.5 mg/kg and 5 mg/kg) was injected intradermally at the base of the tail of each mouse on E0.5d. The mice in IFA-treated group were injected 100μL intradermally at the base of the tail on E0.5.

The mice in the L-NAME group were treated with continuous administration of 125 mg/kg/d of L-NAME, a common vasoconstrictor, starting at 10.5 days of gestation *via* subcutaneous injection on the nucha (18). Control mice were not injected with any solution. Systolic blood pressure (SBP) was recorded every four days. All mice were sacrificed, and placental tissues were harvested on gestational day 17.5 (E17.5d). The numbers of fetus were counted and recorded. We cut open the uterus along the uterine membrane to remove the fetus. Then, we washed the fetus with PBS and placed into a clean dish. The excess liquid was removed by filter paper before weighting.

### Measurements of Blood Pressure in Mice

Monitor of blood pressure were to evaluate the preeclampsia symptom of animal. We determined the blood pressure in conscious mice by tail cuff plethysmography using the Softron BP 2010 (Softron Biotechnology, Beijing, China). All the mice were habituated to the measurement procedure 10 times a day before caging with male mice. At least 9 consecutive measurements were recorded, but only when the condition of the mice was stable. Six time point (pre-pregnancy, E0.5, E4.5, E8.5, E12.5, and E16.5) were selected for assessing systolic blood pressure level throughout the pregnancy. Serial blood pressure measures analyzed by two-way repeated measures ANOVA.

### Cell Culture

HTR-8SV/neo cells (purchased from ATCC, Rockville, USA)were cultured in RPMI-1640 medium containing 10% fetal bovine serum (FBS) and 1% Penicillin-Streptomycin. Cells were maintained at 37°C, with 5% CO_2_. Liguid were changed every other day until cells reached 90% confluence.

### Precoating of Cell Culture Plates With Collagen I

The surfaces of six-well cell culture plates were coated with different doses of collagen I, which was diluted in a minimal volume (500 μL per well). After 4 hours of incubation with collagen I, the solution was removed from the plate surface; plates were then dried overnight at room temperature and washed with phosphate buffered saline to remove the unattached protein.

### Cell Stimulation

HTR-8SV/neo cells were seeded in six-well plate which were incubated with different concentration of collagen I for 48h. CCK-8 (ab228554, Abcam, MA, USA) assay was conducted to detect cell viability and cell cycle analysis cell cycle stage was analyzed using flow cytometry. Western blotting was used to measure the relative protein level. The experimental procedures were detailed in the **Supplementary Material**.

Honokiol, which enhance the phosphorylation of ERK phosphorylation, (HY-N0003, MedChemExpress, USA) dissolved at a concentration of 10μM. In the co-treatment group, HTR-8/SVneo cells were treated with Honokiol and collagen I 48h. SKL-2001 (HY-101085, MedChemExpress, USA), an agonist of β-catenin which was used at a concentration of 5μM. Co-treament with collagen I 24h. Cell viability, cell cycle and relative protein level were analyzed by CCK-8, cycle cell and western blotting.

### Protein Expression and Biochemical Analysis

Protein was extracted from cells and placental tissue with commercial lysis buffer RIPA (89900, Thermo Scientific, USA). Western blotting was performed as described in **Supplementary Material**. Primary antibodies against collagen I (ab260043, Abcam, Cambridge, UK), E-cadherin (#14472, Cell Signaling, USA), N-cadherin (#13116, Cell Signaling, USA), MMP-9(ab38898, Abcam, Cambridge, UK), Vimentin (ab92547, Abcam, Cambridge, UK), GAPDH (ab8245, Abcam, Cambridge, UK), ERK (#4695, Cell Signaling, USA), p-ERK(#4370, Cell Signaling, USA), β-Catenin (ab32572, Abcam, Cambridge, UK).

### Transwell Assay

Transwell assays were performed to test the invasion ability of HTR-8/SVneo Matrigel (356234, BD, USA) and Transwell (3422,Costa, USA) were purchased and used as per manufacturer’s instructions (19). We precoated the upper chamber of each Transwell (Costa, USA) insert with different doses of collagen I (100 or 1000 μg/mL). Cells were grown in the upper chamber in medium containing 3% FBS and assessed for their invasion ability through collagen I toward a chemoattractant (10% FBS) in the lower chamber for 48 h.

### Transcriptome Sequencing and Data Analysis

Differential gene expression analysis of 30,945 expressed genes was performed using DESeq2 (19, 20), and yielded 1237 differentially expressed genes (an adjusted p value < 0.05). We collected PE-related genes from six meta-analyses (21–26), two microarray-based studies (27, 28), one review paper (29), and one research paper based on RNA-seq (30) published since 2009. A total of 3553 PE-associated genes were obtained. We found 2347 of these PE-related genes to be expressed in our dataset, 227 of which were differentially expressed. KEGG (Kyoto Encyclopedia of Genes and Genomes) pathway enrichment analysis for 227 genes was performed with the clusterProfiler package (**Supplementary Table S2**) (31). A cutoff of adjusted P value < 0.05 was chosen to select the most significantly enriched pathways. Finally, pathview (32) was used to visualize the differentially expressed genes belonging to significantly enriched pathways.

### Statistics

Statistical analyses were performed using Prism 7.0 software (GraphPad Software, Inc. San Diego, CA, USA). After testing for normal distribution, differences among groups were analyzed by two-tailed, unpaired Student’s *t*-tests or one-way analysis of variance (ANOVA) with Tukey’s post-hoc test for multiple comparisons. All data are presented as mean ± standard error of mean (SEM).

Enrichment analyses were performed on the R platform, and a two-tailed Fisher’s exact test was used. Error bars were used to represent the standard error of the fraction, estimated using a bootstrapping method with 100 re-samplings. The data relative to the experimental validations are presented as mean ± SEM of two independent experiments. Comparisons between two independent groups were conducted using Student’s *t*-test. Statistical significance was described as *P < 0.05, or **P < 0.01.

## Results

### Elevated Collagen I Levels in Human Placenta From Women Diagnosed With Preeclampsia

We collected placental samples from 20 patients, 10 of whom were diagnosed with preeclampsia and 10 represented normotensive controls. The characteristics of the study cohort are presented in **Supplementary Table S1**. As expected, increased blood pressure and proteinuria were evident in the preeclampsia-diagnosed group.

**Figure 1:**
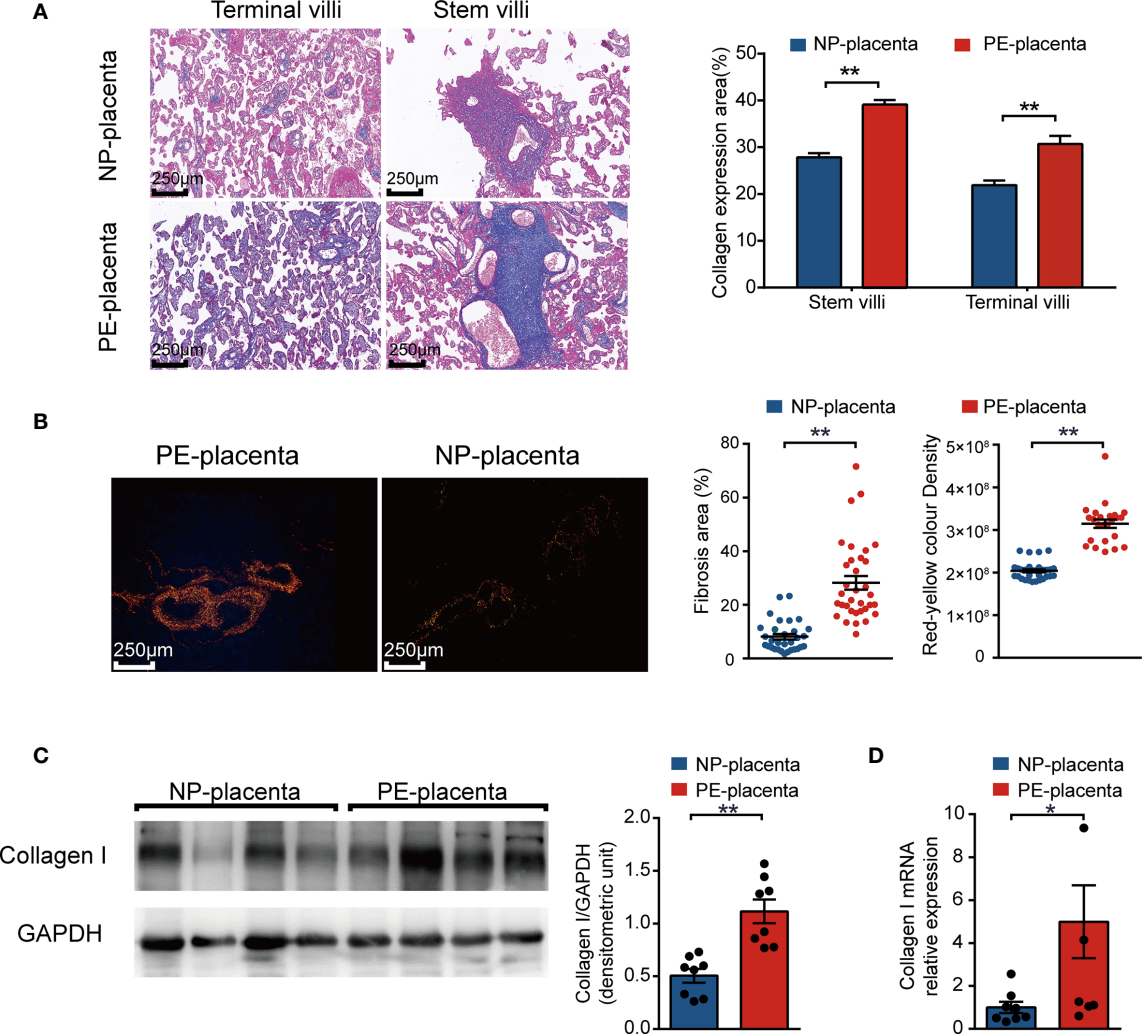
Identification and quantification of collagen types in human preeclamptic (PE) placentas. **(A)** Masson’s trichrome staining of placentas in the stem and terminal villus region from normotensive pregnant women (NP placenta) and from PE patients (PE placenta; n = 6 per group, scale bar = 250 nm). Collagen expression area of PE and NP placentas. Collagen fibers were stained blue, while cytoplasm and red blood cells were stained red, and the nucleus blue and brown. **(B)** Representative image of Sirius red staining of placenta (magnification, 200×). Measured area and red-yellow color intensity of Sirius Red staining in PE and NP placenta (three visual fields for each placental sample, n = 6 per group). **(C)** Representative image of western blotting and quantitative analysis of collagen I in PE and NP placenta (bar chart, n = 8 per group). **(D)** Relative gene expression of collagen I (bar chart, n = 8 per group). Data are presented as mean ± standard error of mean (SEM). *p < 0.05, **p < 0.01 by two-tailed unpaired *t*-test analysis.

The degree of fibrosis of the various placental samples is shown in **Figure 1A**. Collagen deposited in villous tissues and wrapped in blood vessels. According to the Masson’s trichome staining the collagen expression, a high degree of fibrosis of stem villi and terminal villi was more frequent in preeclamptic placenta. Sirius Red staining is a method used for collagen identification. The elongated axis of dye molecules are attached parallel to collagen, resulting in enhanced birefringency and specificity when combined with polarized light detection methods (33). Collagen I appears red-yellow when observed under a polarizing microscope (34). Notably, the red-yellow color density was much higher in preeclamptic placentas than in control placenta (**Figure 1B**). Further, the relative gene and protein expression levels of collagen I were higher in preeclamptic placenta (**Figures 1C, D**). These results indicated that collagen I may be the characteristic collagen deposited in human preeclamptic placenta.

### Collagen I Induces Preeclampsia-Like Symptoms in Pregnant Mice

**Figure 2:**
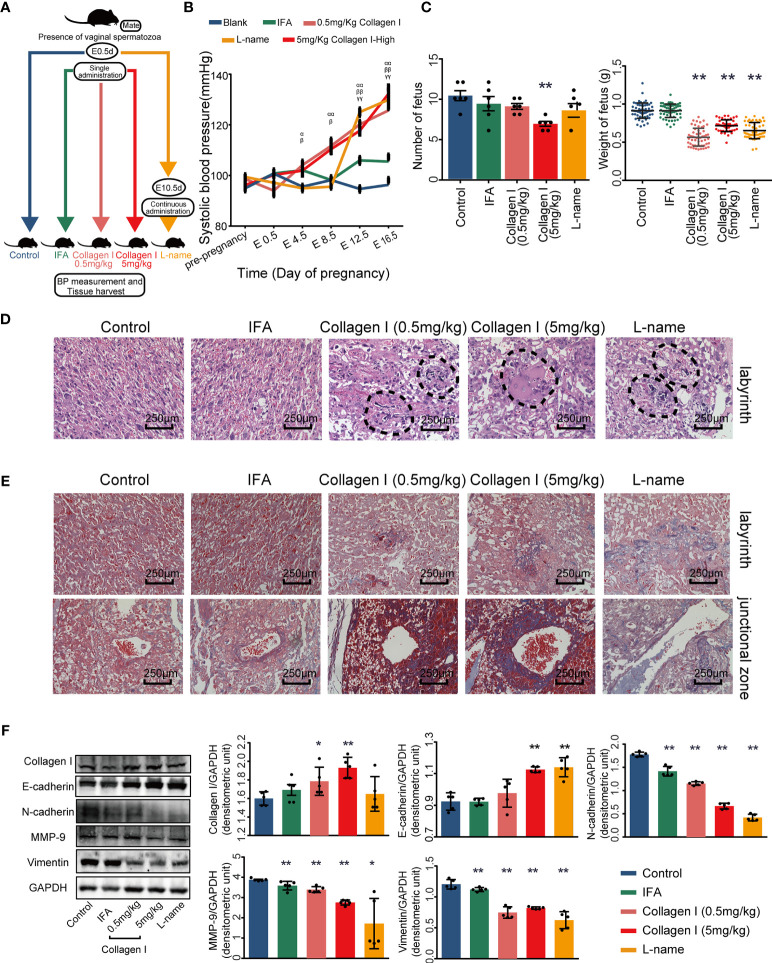
Collagen I induces preeclampsia-like symptoms in mice. Effects of collagen I on the reproductive outcomes of pregnant mice. **(A)** Schematic representation of the experimental groups. **(B)** The systolic blood pressure of members of the five groups during pregnancy (n = 6 per group, α indicates mice treated with 5 mg/kg of collagen I *vs.* control, β indicates mice treated with 0.5 mg/kg of collagen I *vs.* control, γ indicates L-NAME-treated mice *vs.* control, with ^α,β,γ^p < 0.05 and ^αα,ββ,γγ^p < 0.01, according to two-way repeated measures ANOVA). **(C)** The number of offspring significantly decreased in the group treated with 5 mg/kg of collagen I (n = 6 per group) compared with the control at 17.5 days of gestation. Fetal weights in the groups treated with 0.5 mg/kg of collagen I (n = 54), 5 mg/kg of collagen I (n = 46), and L-NAME (n = 49) were significantly decreased compared with those of the control (n = 59) and IFA-treated groups (n = 56). **(D)** Representative image of the H&E-stained labyrinth zone of mouse placenta. Typical lesions are marked with dotted lines (original magnification, ×200; scale bar = 250 μm, n = 4). **(E)** Representative image of Masson’s trichrome staining in the labyrinth and junctional zone of the mouse placenta. (original magnification, ×200; scale bar = 250 μm, n = 4). **(F)** Western blot analysis of the protein level of collagen I and the proteins related to PE pathogenesis including MMP-9, E-cadherin, N-cadherin, and vimentin (n = 5, asterisks indicate differences between groups *vs.* control, with *p < 0.05, **p < 0.01, according to one-way ANOVA followed by Dunnett post-hoc test).

To further test whether excessive collagen I can cause PE progression *in vivo*, pregnant mice were randomly divided into five groups on E0.5d (**Figure 2A**).

At E4.5 d, collagen I-injected mice exhibited significantly higher SBP than did control and IFA mice (p < 0.05). Moreover, the SBP of the collagen I- and L-NAME-treated groups was elevated from the second trimester to the end of gestation (p < 0.01 **Figure 2B**). Placental tissue was harvested at E17.5d to examine the weight of foetuses and the structure of the placenta. Fetal weight was decreased in both the collagen I- and L-NAME-treated groups (p < 0.01 **Figure 2C**). However, the number of fetus only decreased in the group treated with 5 mg/kg of collagen I (p < 0.01 **Figure 2C**). Nevertheless, histomorphometric analysis revealed structural changes in the placenta of mice in the collagen I- and L-NAME-treated groups; particularly, infarction was observed in the labyrinth layer (**Figure 2D**). Moreover, in both the labyrinth and junctional zones, a higher degree of fibrosis was observed in the collagen I- and L-NAME-treated groups than in the control group (**Figure 2E**). However, the relative protein expression of collagen I was higher only in the collagen I-treated groups (p <0.01 in the 5mg/kg group and p <0.05 in the 0.5mg/kg group, **Figure 2F**). These results indicated that exposure to collagen I injection during early pregnancy may result in an increased deposition of collagen I in the placenta, and in subsequent preeclampsia-like adverse reproductive outcomes.

In addition to histological changes, defective trophoblast invasion is involved in the pathogenesis of preeclampsia (35). Therefore, we analyzed the relative protein expression of MMP-9, E-cadherin, N-cadherin, and vimentin, which regulate trophoblast invasion (36–38), using western blotting. Upon exposure to collagen I, the relative protein expression of MMP-9, N-cadherin, and vimentin in the placenta decreased compared to that of the control placenta, while E-cadherin expression increased. These results suggested that collagen I induced preeclampsia-like symptoms by suppressing the invasive ability of trophoblasts.

### Collagen I Suppress the Proliferation and Invasive Ability of HTR-8SV/neo Cells

**Figure 3:**
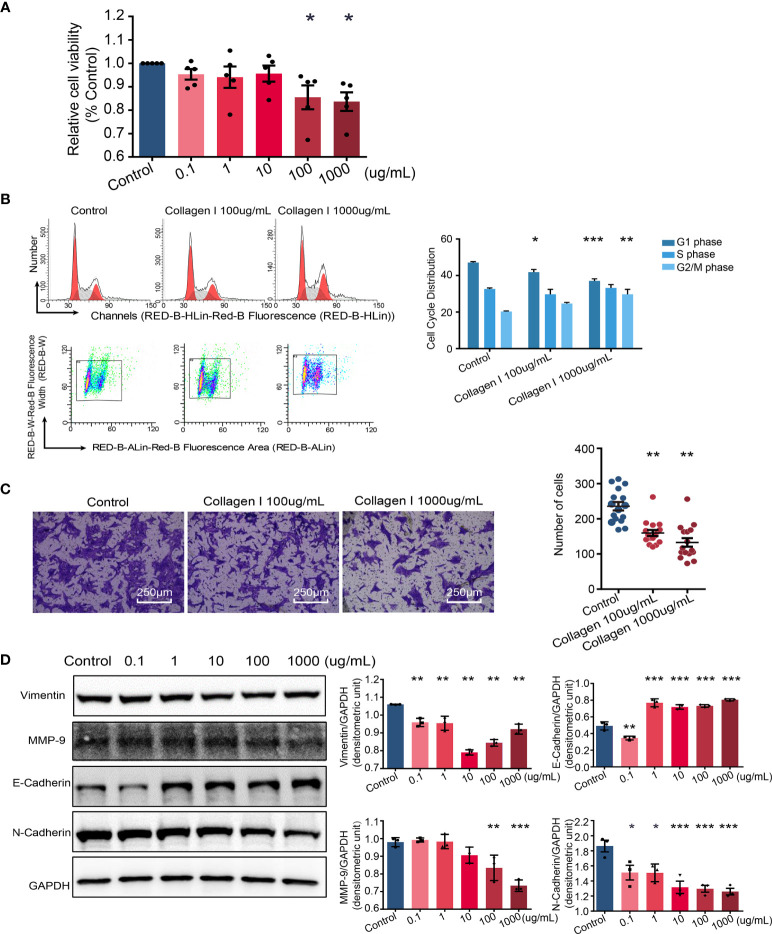
Collagen I induce trophoblast dysfunction in HTR-8/SVneo cells. Effect of collagen I on proliferation and invasion abilities of HTR-8/SVneo cells. **(A)** HTR-8/SVneo cells were cultured at different concentrations of collagen I (0 μg/mL, 0.1 μg/mL, 1 μg/mL, 10 μg/mL, 100 μg/mL, and 1000 μg/mL). Cell proliferation of HTR-8SV/neo cells was analyzed by CCK-8 assay after 48 hours of incubation (n = 5 per group). **(B)** Effect of collagen I on the cell cycle profile of HTR-8SV/neo cells. After cultured at 100 μg/mL and 1000 μg/mL of collagen I for 48 hours, cells were labeled with propidium iodide and the stain was detected by laser scanning cytometry(LSC). Bar plot representing the proportion of cells at each phase of the cell cycle (G1, S, and G2/M phases) in three different cell groups (cells grown in presence of 100 μg/mL or 1000 μg/mL of collagen I and control cells; n = 5 per group). **(C)** Migration of HTR-8/SVneo cells. The total number of invading cells was counted in four representative fields under ×200 magnification (n = 4 per group). **(D)** Representative western blot image and quantitative analysis of vimentin, MMP-9, E-cadherin, and N-cadherin expression in the three cell groups (n = 3 per group). Asterisks indicate differences between groups *vs.* control, with *p < 0.05, **p < 0.01, and ***p < 0.001, according to one-way ANOVA followed by Dunnett post-hoc test.

Defective trophoblast proliferation and invasive ability are important factors contributing to pathogenesis of preeclampsia (35). At collagen I doses of 100 and 1000 µg/mL, the viability of cells decreased compared with that of cells grown in control plates (p < 0.05 **Figure 3A**). Particularly, collagen I significantly induced cell cycle arrest at the G2/M phase at a dose of 1000 µg/mL (p < 0.01 in the 1000µg/mL group, **Figure 3B**). Further, cell invasion was significantly attenuated by collagen I (p < 0.01 **Figure 3C**). Because MMP-9, E-cadherin, N-cadherin, and vimentin have been implicated in preeclampsia pathogenesis, we investigated their relative expression by western blot analysis and found that collagen I significantly decreased MMP-9, vimentin, and N-cadherin expression, but increased E-cadherin protein levels (**Figure 3D**).

Altogether, our *in vitro* experiments suggest that collagen I induced trophoblast dysfunction by suppressing the proliferation and invasive ability of this cell type.

### Collagen I Affect Gene Expression in Trophoblasts

**Figure 4:**
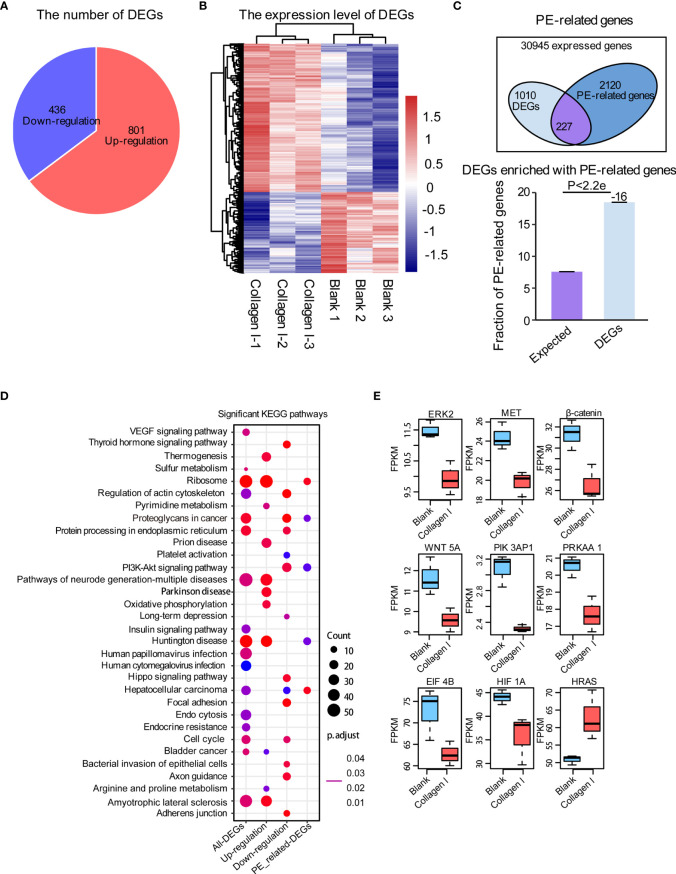
Collagen I induce transcriptional changes in HTR-8SV/neo cells. Overview of differentially expressed genes upon collagen I treatment of HTR-8SV/neo cells. **(A)** Pie chart showing the number of differentially expressed genes between collagen I-treated cells and the control group. **(B)** Heatmap showing the expression levels of differentially expressed genes. **(C)** Venn diagram showing the PE-related genes expressed in our dataset. Differentially expressed genes were enriched in PE-related genes (p < 0.05). **(D)** Significantly enriched KEGG pathways within total differentially expressed genes, including upregulated genes, downregulated genes, and differentially expressed PE-related genes. **(E)** Boxplots showing the relative expression levels of *ERK2 (MAPK1)*, *MET*, *β-catenin, WNT5A*, *PIK3AP1*, *PRKAA1*, *EIF4B*, *HIF1A*, and *EIF4B*. Enrichment analyses were performed on the R platform, and two-tailed Fisher’s exact test was used. Error bars represent the standard error of the fraction, estimated using a bootstrapping method with 100 re-samplings. The data of the experimental validations are presented as mean ± standard error of mean (SEM) of two independent experiments. Comparisons between two independent groups were conducted using Student’s *t*-test.

To identify collagen I-dependent changes in gene expression patterns of trophoblasts at the transcriptional level, we analyzed total RNA samples from 100μg/mL collagen I-treated and untreated HTR-8SV/neo cells by RNA-seq. We found that the expression of 801 genes increased while that of 436 genes decreased under collagen I treatment compared with gene expression in the control group (**Figure 4A**). The relative expression of upregulated and downregulated genes was illustrated in a heat map (**Figure 4B**). Additionally, we found 2347 PE-related genes to be expressed in our dataset, among which 227 were differentially expressed (**Figure 4C**). With the latter gene set, we carried out KEGG pathway enrichment analysis; the significantly enriched pathways are summarized in **Figure 4D**. Interestingly, genes involved in proteoglycans in cancer, cell cycle, and the PI3K-AKT signaling pathway (which is related to cell proliferation and invasion), were significantly downregulated in our dataset. Consistently, collagen I treatment downregulated the expression of *ERK2, MET, PI3K, β-catenin*, and *WNT5A*, genes related to cell proliferation and invasion in trophoblasts (**Figure 4E**).

### Collagen I Suppress Trophoblast Proliferation and Invasion

**Figure 5:**
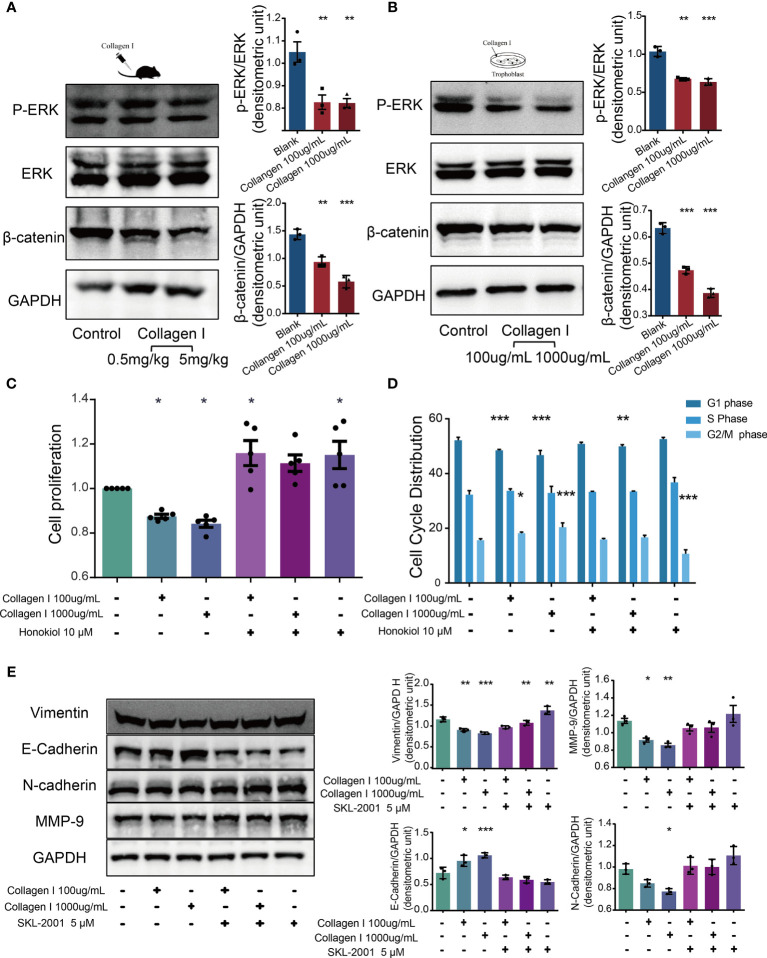
Collagen I suppressed proliferation and invasion of trophoblasts through inhibition of ERK phosphorylation and the WNT/β-catenin signaling pathway. Honokiol and SKL-2001 reversed the decrease in proliferation and invasion ability of trophoblasts induced by collagen I. **(A)** Representative western blot image and quantitative analysis of p-ERK, ERK, and β-catenin levels in collagen I-treated mouse placentas (n = 3 per group). **(B)** Representative western blot image and quantitative analysis of p-ERK, ERK, and β-catenin levels in collagen I-treated HTR-8/SVneo cells (n = 3 per group). **(C)** After incubation with honokiol and collagen I for 48 hours, HTR-8/SVneo cell proliferation was measured by CCK-8 assay (n = 5 per group). **(D)** After incubation with honokiol and collagen I for 48 hours, cells were labelled with propidium iodide (PI) and the stain was detected by LSC. Bar plots representing the proportion of cells at each phase of the cell cycle (G1, S, and G2/M phases) in three different cell groups (n = 5 per group). **(E)** Representative western blot image and quantitative analysis of MMP-9, vimentin, E-cadherin, and N-cadherin levels in collagen I- and/or SKL-2001-treated HTR-8/SVneo cells (n = 3 per group). Asterisks indicate differences between groups *vs.* control, with *p < 0.05, **p < 0.01, and ***p < 0.001, according to one-way ANOVA followed by Dunnett’s post-hoc test.

Together with transcriptome sequencing analysis, western blotting was used to detect p-ERK/ERK and β-catenin expression in collagen I-treated and untreated mouse placenta and HTR-8SV/neo cells. In both types of samples, we observed decreased expression of p-ERK and β-catenin after collagen I treatment (**Figures 5A, B**). ERK is a member of the MAPK signaling pathway. Alterations in ERK phosphorylation can lead to changes in cell proliferation (39). The present results demonstrated that the ERK phosphorylation level was decreased following treatment with collagen I. Furthermore, the combination of the ERK activator, honokiol, with collagen I to treat HTR-8/SVneo cells, reversed the decline in proliferation and the G2/M arrest induced by collagen I (**Figures 5C, D**).

Wnt/β-catenin signaling is an essential pathway promoting blastocyst activation and implantation. In particular, β-catenin plays a central role in canonical Wnt/β-catenin signaling. β-catenin levels decreased in both collagen I-treated mouse placenta and HTR-8/SVneo cells. Therefore, SKL-2001, an agonist of the Wnt/β-catenin pathway that can stabilize intracellular β-catenin levels, was used to clarify the role of β-catenin in collagen I-dependent remodulation of trophoblast properties. The relative protein levels of MMP-9, vimentin, N-cadherin, and E-cadherin were also recovered compared with those of collagen I-treated cells (**Figure 5E**).

Overall, our results showed that activators of ERK and β-catenin (honokiol and SKL-2001) reversed the decline in cell proliferation and invasion induced by collagen I, demonstrating that collagen I suppressed proliferation and invasion through inhibition of ERK phosphorylation, and the Wnt/β-catenin signal pathway.

## Discussion

**Figure 6:**
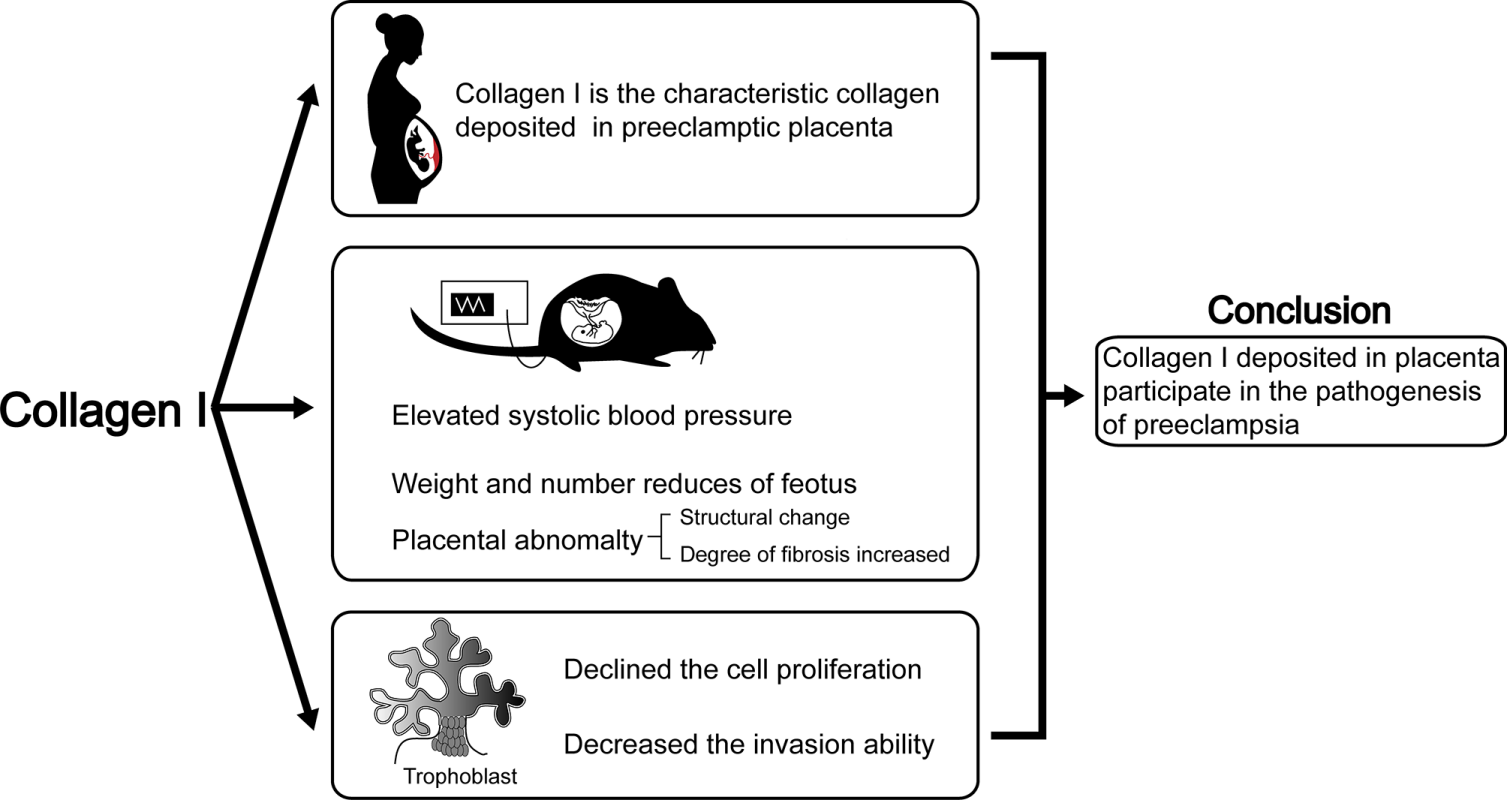
The abstract graphic. Collagen I deposited in placenta participate in the pathogenesis of preeclampsia: Collagen I is the characteristic collagen deposited in preeclamptic placenta. Collagen I administration is associated with suppressed proliferation and invasion of trophoblasts, and with preeclampsia-like symptoms in pregnant mice.

Herein, we proved the characteristic role of Collagen I in preeclamptic placenta is consistent with previous reports (17). Moreover, we determined that excess collagen I accumulation is associated with preeclampsia-like symptoms in pregnant mice. Indeed, a single injection of collagen I in early pregnancy led to increased blood pressure on E4.5d, which continued to stay elevated until term. Furthermore, the weight and number of offspring decreased when the tissue was harvested at term. This phenomenon might depend on the decreased proliferation and invasion ability of trophoblasts in the presence of excess collagen I. In summary, we propose that excessive collagen I deposition in the placenta plays a crucial role in preeclampsia pathogenesis (summarized in **Figure 6**).

The pathogenesis of preeclampsia is still unclear. A possible reason for this is the difficulty in performing high-quality research on the etiology of placental pathologies in humans because of ethical challenges associated with the examination of pregnant women in the first trimester. Thus, animal models of preeclampsia are the most important tool for longitudinal investigation of adverse pregnancy outcomes (40). For instance, treating third-trimester pregnant mice with L-NAME, a common vasoconstrictor, to induce PE symptoms is a common practice, as once drug treatment is stopped, blood pressure returns to normal (41). In our study, a single injection of collagen I at early pregnancy led to changes in placental structure at term. In addition to a higher degree of fibrosis, collagen I-treated placentas showed a significantly higher collagen I protein level than the control group. Moreover, preeclampsia-like symptoms were induced in later trimesters of pregnancy. In comparison with L-NAME treatment, the symptoms induced by collagen I did not depend on continuous medication, but occurred in the second trimester and continued through the third. These disease dynamics were close to those observed in clinical practice.

It is widely accepted that primary defective trophoblast invasion, leading to inadequate transformation of maternal uterine vasculature, probably drives preeclampsia pathogenesis. It has also been indicated that early pregnancy placentation is closely related to trophoblast function and behavior (42). In our *in vitro* study, excessive collagen I induced trophoblast dysfunction by direct stimulation. It decreased the proliferation and invasion of HTR-8/SVneo cells. Surprisingly, we found that preeclampsia-like symptoms emerged in later pregnancy and infarction and a high degree of fibrosis were observed in the placenta after a single injection of collagen I on E0.5 d *in vivo*. Moreover, changes in MMP-9, vimentin, E-cadherin and N-cadherin expression both in placental and HTR-8SV/neo cells were consistent with the phenotypes of preeclampsia (36–38) after treatment with collagen I. Thus, we suggest that the single injection of collagen I in early pregnancy induced preeclampsia development by interfering with placentation in early pregnancy due to impaired trophoblast invasion. Collagen I is the most abundant structural protein in most tissues (43). However, excessive accumulation of collagen I may lead to fibrosis, which in turn impairs normal organ function. Trigonelline hydrocholoride, targeting collagen I fibrillation, could ameliorate fibrosis of the myocardium (44). Therefore, collagen I may also be a novel target for therapeutic interventions in patients with preeclampsia.

The ERK/MAPK pathway is an important signaling cascades that is involved in cell proliferation and embryo development (45). We found that collagen I downregulated ERK phosphorylation in HTR-8/SVneo cells and mouse placenta compared with the control. Honokiol which enhances the phosphorylation of ERK phosphorylation, could reverse the weakened proliferation induced by collagen I. Further, the canonical “Wnt/β-catenin” pathway regulates cell invasion, and abnormal Wnt/β-catenin expression contributes to preeclampsia development (46). β-catenin and GSK-3β were proteins levels were reportedly significantly decreased in severe preeclamptic placenta (47). In our study, β-catenin expression in collagen I-treated placentas was less than that in the pregnant control Transcriptome analysis suggested β-catenin and GSK-3β expression decreased in HTR-8/SVneo cells incubated with 100µg/mL collagen I. To verify these findings, SKL-2001, an agonist of that β-catenin was used. *In vitro*, SKL-2001 could reverse the downregulation of MMP-9, N-cadherin and vimentin induced by collagen I. Thus, the results suggested that collagen I suppresses trophoblast proliferation and invasion through inhibition of ERK phosphorylation, and the WNT/β-catenin signaling pathway.

There were some limitations of this study. First, our experiments demonstrated that the dysfunction of HTR-8/SVneo cells induced by collagen I can be reversed by ERK and β-catenin agonists. Therefore, the possible role of upstream regulators of the ERK/MAPK and WNT/β-catenin pathways in preeclampsia pathogenesis should be investigated. Second, placental Masson’s and Sirius red staining showed that the degree of fibrosis in PE patients was more serious than that in normal patients after delivery. However, whether the extent of placental fibrosis and its alteration can be determined by ultrasound or other diagnostic methods during pregnancy, calls for further clinical study and discussion. Third, small-molecule inhibitors against placental collagen I deposition are still lacking and should be explored as a future research avenue. Finally, the sample is relatively small, larger sample size is required in our coming study.

Overall, in this study we confirmed that the preeclamptic placenta shows significant histological signs of fibrosis and is characterized by collagen I deposition. Furthermore, an excess of collagen I caused preeclampsia-like features in pregnant mice by suppressing proliferation and invasion of trophoblasts. *In vitro*, we detected altered expression of genes related to proliferation and invasion upon collagen I stimulation. In conclusion, our study points at the relevant role of collagen I in the development of preeclamptic placentas, providing new insights into the pathogenesis of preeclampsia.

## Data Availability Statement

The datasets presented in this study can be found in online repositories. The names of the repository/repositories and accession number(s) can be found below: https://data.mendeley.com/datasets/hcd2c3n9tc/1, The Mendeley dataset DOI: 10.17632/hcd2c3n9tc.1. https://github.com/YingLin-Feng/Collagen-I-and-preeclampsia.

## Ethics Statement

The collection of placentae was approved by the Ethics Committee of Nanfang Hospital (NFEC-2017-055). All participants gave written consent prior to donating their placenta. The patients/participants provided their written informed consent to participate in this study. This project was performed in accordance with animal protocol procedures approved by the Department of Laboratory Animal Sciences, Southern Medical University (L-2019216).

## Author Contributions

YF, XiC, and LH designed the study. Data collection was performed by HW, YiC, ML, ZL, XuC, YuC, YW, CS, and YH. Data analysis was done by PL, JL, MZ, ZW, and XY. YF wrote the manuscript and designed the figures, whilst all other authors revised the manuscript. All authors contributed to the article and approved the submitted version.

## Funding

This work was funded by the National Natural Science Foundation of China (82071669), Foshan Dengfeng project(2020B002), the Natural Science Foundation of Guangdong Province (2019A1515010637) and Science and Technology Planning Project of Guangdong Province (2017A010105025).

## Conflict of Interest

The authors declare that the research was conducted in the absence of any commercial or financial relationships that could be construed as a potential conflict of interest.
