# Supplementary material for: Collagen I Induces Preeclampsia-Like Symptoms by Suppressing Proliferation and Invasion of Trophoblasts
Source: Front Endocrinol (Lausanne). 2021 Aug 6;12:664766. doi: 10.3389/fendo.2021.664766 (PMC8378400; doi:10.3389/fendo.2021.664766)
Supplement: Supplementary file 1 [file DataSheet_1.pdf]

Supplemental Materials for

Collagen I induces preeclampsia-like symptoms by suppressing proliferation and invasion of trophoblasts

Yinglin FENG[1†], Xia CHEN[2†], Huiqiao WANG[3], Xueping CHEN[1], Zixin LAN[3],Pan LI[4],Yingshi CAO[1], Mian LIU[1],Jin LV[5], Yun CHEN[1],Yu WANG[1],Chao SHENG[1], Yingying HUANG[1], Mei ZHONG[1],Zhijian WANG[1],Xiaojing YUE[1*],Liping HUANG[1*]

[1.]Department of Obstetrics and Gynecology, Nanfang Hospital, Southern Medical University, Guangzhou, China

[2.]Department of Obstetrics and Gynecology, Foshan First People's Hospital, Foshan, China

[3.] Zhujiang Hospital, Southern Medical University, Guangzhou, China.

[4.] Microbiome Research Center, University of New South Wales, Australia

[5.] Department of Pathology, Foshan First People's Hospital, Foshan, China

Short title: Collagen I in preeclampsia

***Contributed equally.**

Corresponding author: Professor Liping Huang and Xiaojing YUE, Department of Obstetrics and Gynecology, Nanfang Hospital, Southern Medical University, Guangzhou 510515, China; Tel: +86(20)61641909

This file includes：

Expanded Materials & Methods

Supplemental tables S1, S2

Expanded Materials & Methods

Placental histological analysis hematoxylin eosin (H&E), Masson-trichrome and

Sirius red staining

After fixation in 4% paraformaldehyde of placenta, the slices were embedded in paraffin. Section of 3μm were stained by structural identification, with Masson's trichrome staining for collagen fiber observation, and with Sirius red stain for collagen identification.

Masson-trichrome staining

For Masson trichrome staining, the sections were stained using a Masson's trichrome staining kit (Sigma-Aldrich, St. Louis, MO, USA) according to the manufacturer's protocol. The slides were incubated in Weigert's iron hematoxylin (5 min), Biebrich Scarlet-Acid Fuchsin Solution (15 min), Phosphomolybdic- Acid Solution (15 min) and Aniline Blue Solution (10 min), all at room temperature. The slides were visualized under the light microscope. Collagen fiber were stained blue, while cytoplasm and red blood cells were stained red and nucleus blue and brown. Fibrosis area% was calculated in μm digitally using the software NDP.view2 (Hamamatsu Corp, Japan). The area of tubulointerstitial fibrosis was measured in 5 random fields under ×200 magnification.

Sirius red staining

For Sirius Red staining, the Picrosirius Red stain kit (Abcam, Cambridge, UK) was utilized. Sections were stained with Weigert's iron Sumu essence dye for 15 min, rinsed for 5 min, and then washed with distilled water. The sections were covered with 200 μl Sirius red dye for 1 h. Each analyzed field was chosen randomly and the positive red-stained areas and red-yellow density were quantified using computerized image

analysis software (NIH, MD, USA).

Western blotting

Placenta tissue and *in vitro*-treated cells was homogenized and protein extracted as described below.

Homogenized by a Qiacube machine (Qiagen, Courtaboeuf, Fance) in RIPA lysis buffer (Thermo, Rockford, USA) on ice. The supernatants were collected after centrifugation at 12000×g at 4°C for 20 min. Protein concentration was determined using a BCA protein assay kit (Thermo, Rockford, USA), and whole lysates were mixed with 5×SDS loading buffer (Coolaber, Shanghai,China) at a ratio of 1:4. Protein samples were heated at 98°C for 5 min and were separated on SDS-polyacrylamide gels(Biodragon,Guangzhou,China). The separated proteins were then transferred to a PVDF membrane (Dogesce, Shanghai, China). The membrane blots were first probed with a primary antibody. After incubation with horseradish peroxidase-conjugated second antibody, autoradiograms were prepared using the enhanced chemiluminescent system to visualize the protein antigen. The signals were recorded using SYNGENE BIO IAMGING (GENE GNOME, Shanghai,China). Primary antibodies for Western Blot are rat anti-collagen I, anti-MMP9, anti-, anti-vimentin, anti-E-cadherin, anti-N-cadherin, anti-β-catenin, anti-ERK, anti-p-ERK and rabbit anti-GAPDH (Cell Signaling, San Jose, CA, USA). GAPDH was used as a protein loading control. The secondary antibody was HRP-conjugated anti-rabbit (Cell Signaling, San Jose, CA, USA). Images shown in the figures were representative of 5 individuals. ImageJ software (https://imagej.nih.gov/ij) was used for image acquisition and densitometric

analysis of the gels.

Quantitative PCR (RT-qPCR)

Total RNA was extracted with Trizol reagent (Invitrogen, Corporation, USA) according to the manufacturer’s instructions. The reverse transcription reaction was carried out with reverse transcription enzyme (Toyobo, Shanghai, China). Quantitative real-time PCR was carried out on an LightCycler96 real-time PCR system (Roche,Basel, Switzerland) and the specific primers for quantitative PCR are shown below (IGE,Guangzhou,China).

**Collagen I**	Forward:	5′-CCAAGACGAAGACATCCCACCA-3′
	Reverse:	5′-CCGTTGTCGCAGACGCAGAT-3′

CCK-8

HTR-8/SVneo cells were seeded in 96-well plates at the density of 10000 cells per well in 250μl of complete culture medium. After treatments, three methods were utilized for cell proliferation analysis. Cell Counting Kit-8 (Beyotime, Guangzhou, China) analysis: 10 ul of CCK-8 was added to each well. The culture plates were shaken for 90 min and the optical density (OD) values were read at 450 nm.

Cell cycle

After treatment of collagen I, HTR-8SV/neo cells were harvested by trypsinization and washed with cold PBS for two times. Then, cells were fixed with 75% alcohol for 12 h at 4°C. After washing with cold PBS, cells were treated with 50 ug/mL RNase (MULTI SCIENCES, Shanghai, China) for 30 min at 37 °C, then stained with 50 μg/mL Propidium iodide(MULTI SCIENCES, Shanghai, China) for 30 min at 4 °C in the

dark before being analyzed using a FACS Calibur flow cytometer (BD,CA, USA). Cells (1×10^6) were detected for each sample. Cell cycle was analyzed by ModFit software (Verity Software House,ME,USA).

Transcriptome sequencing

Sample collection and preparation

HTR-8SV/neo cells were cultured in the medium described above in plates precoated with 100 μg/mL of collagen I for 48 hours. Total RNA was extracted using Trizol. After total RNA was extracted, Protential RNA degradation and contamination was monitored on 1% agarose gel. RNA purity was checked using the NanoPhotometer® spectrophotometer (IMPLEN, CA, USA) and RNA integrity was assessed using the RNA Nano 6000 Assay Kit of the Bioanalyzer 2100 system (Agilent Technologies, CA, USA).

Library preparation for Transcriptome sequencing

A total amount of 1 μg RNA per sample was used as input material for the RNA sample preparations. Sequencing libraries were generated using NEBNext® UltraTM RNA Library Prep Kit for Illumina® (NEB, Massachusetts,USA) following manufacturer's recommendations and index codes were added to attribute sequences to each sample.

Briefly, mRNA was purified from total RNA using poly-T oligo-attached magnetic beads. Fragmentation was carried out using divalent cations under elevated temperature in NEBNext First Strand Synthesis Reaction Buffer(5X). First strand cDNA was synthesized using random hexamer primer and M-MuLV Reverse

Transcriptase(RNase H-). Second strand cDNA synthesis was subsequently performed using DNA Polymerase I and RNase H. Remaining overhangs were converted into blunt ends via exonuclease/polymerase activities. After adenylation of 3’ ends of DNA fragments, NEBNext Adaptor with hairpin loop structure were ligated to prepare for hybridization. In order to select cDNA fragments of preferentially 250~300 bp in length, the library fragments were purified with AMPure XP system (Beckman Coulter, Beverly, USA). Then 3 μl USER Enzyme (NEB, USA) was used with size-selected, adaptor-ligated cDNA at 37°C for 15 m in followed by 5 min at 95°C before PCR. Then PCR was performed with Phusion High -Fidelity DNA polymerase, Universal PCR primers and Index (X) Primer. At last, PCR products were purified (AMPure XP system) and library quality was assessed on the Agilent Bioanalyzer 2100 system.

Clustering and sequencing

The clustering of the index-coded samples was performed on a cBot Cluster Generation System using TruSeq PE Cluster Kit v3-cBot-HS (Illumia, CA, USA) according to the manufacturer’s instructions. After cluster generation, the library preparations were sequenced on an Illumina Novaseq platform and 150 bp paired-end reads were generated.

Supplemental Materials On line table

Table S1 Comparison of clinical data in two study group

Table S1 Characteristics of the study cohort (Mean ± SEM)

Characteristic	Normal	Preeclampsia	p-value
N	10	10	
Age(y)	30.40 ± 0.96	33.10 ± 2.17	p=0.27
SBP (second trimester, mmHg)	120.9 ± 2.82	158.6 ± 3.29	p<0.01
DBP (second trimester, mmHg)	73.90 ± 1.73	99.70 ± 3.25	p<0.01
MAP (second trimester, mmHg)	89.56 ± 2.09	118.73 ± 3.26	p<0.01
Gestational weeks,（w）	39.53 ± 0.28	35.66 ± 0.86	p<0.01
Body Mass Index（kg/cm2)	27.48 ± 0.94	29.68 ± 1.59	p=0.39
ALT (third trimester, U/L)	15.30 ± 3.43	11.20 ± 1.54	p=0.29
AST (third trimester, U/L)	18.80 ± 1.02	18.80 ± 2.21	p=1
albumin (third trimester, g/L)	35.58 ± 1.034	38.55 ± 0.3859	p<0.05
Total protein (third trimester, g/L)	64.96 ± 1.349	63.12 ± 1.075	p=0.30
Hb (third trimester, g/L)	108.2 ± 2.01	121.4 ± 3.43	p<0.01
PLT (third trimester, *10^9NAl)	222.7 ± 17.81	242.3 ± 21.15	p=0.49
Cr (third trimester, µmol/L)	47.20 ± 2.78	56.79 ± 3.71	p=0.05
Bun (third trimester, mmol/mL)	3.54 ± 0.26	4.16 ± 0.52	p=0.30
D-Dimer (third trimester, ug/mL)	0.75 ± 0.19	0.52 ± 0.11	p=0.32
Proteinuria(0 ~ ++++)	< +	>++	p<0.01
Intrapartum hemorrhage（volume，mL）	249.0±20.96	264.0±39.53	p=0.74
Neonatal weight（g）	3276 ± 0.15	2730 ± 0.28	p=0.11

Cesarean delivery, n (%)	30%	60%
Primiparous, n (%)	40%	50%

Note: Differences in characteristics between PE and Normotension group were evaluated, using Student's t-test.

Abbreviations: N, sample size; Normal, normotensive pregnant women; PE, preeclampsia; SBP, systolic blood pressure; DBP, diastolic blood pressure; MAP, Mean arterial pressure; ALT, alanine aminotransferase; AST, Aspartate aminotransferase; Cr, creatinine; Bun,urea nitrogen; Hb, hemoglobin; PLT, blood platelet.

Evaluation of proteinuria was estimated by examination of fresh urine using multistix (Bayer, Auckland, New Zealand) on a scale of 0 to 4 +,
where 0/trace=negative, 1+ =30, 2+ =100, 3+ =300, and 4+ =over 2000 mg/dl.

Table S2 KEGG (Kyoto Encyclopedia of Genes and Genomes) pathway enrichment analysis

Table S2 KEGG (Kyoto Encyclopedia of Genes and Genomes) pathway enrichment analysis

ID	Description	GeneRatio	BgRatio	pvalue	p.adjust	qvalue	Count	group
hsa03010	Ribosome	48/576	158/8076	8.24E-19	2.47E-16	2.07E-16	48	All-DEGs
hsa05016	Huntington disease	45/576	306/8076	2.11E-06	0.000317	0.000266	45	All-DEGs
hsa05205	Proteoglycans in cancer	31/576	205/8076	4.86E-05	0.004864	0.004079	31	All-DEGs
hsa04141	Protein processing in endoplasmic reticulum	27/576	171/8076	6.99E-05	0.00524	0.004395	27	All-DEGs
hsa00920	Sulfur metabolism	5/576	10/8076	0.000338	0.017164	0.014394	5	All-DEGs
hsa05014	Amis	44/576	364/8076	0.000343	0.017164	0.014394	44	All-DEGs
hsa04110	Cell cycle	20/576	124/8076	0.000449	0.017533	0.014703	20	All-DEGs
hsa05219	Bladder cancer	10/576	41/8076	0.000468	0.017533	0.014703	10	All-DEGs
hsa05022	Pathways of neurodegeneration - multiple diseases	53/576	475/8076	0.000637	0.019157	0.016065	53	All-DEGs
hsa05165	Human papillomavirus infection	40/576	331/8076	0.000639	0.019157	0.016065	40	All-DEGs
hsa04370	VEGF signaling pathway	12/576	59/8076	0.000777	0.021197	0.017776	12	All-DEGs
hsa01522	Endocrine resistance	16/576	98/8076	0.001425	0.035201	0.029519	16	All-DEGs
hsa04910	Insulin signaling pathway	20/576	137/8076	0.00162	0.035201	0.029519	20	All-DEGs
hsa04810	Regulation of actin cytoskeleton	28/576	218/8076	0.001643	0.035201	0.029519	28	All-DEGs
hsa05225	Hepatocellular carcinoma	23/576	168/8076	0.00181	0.035874	0.030084	23	All-DEGs
hsa04144	Endocytosis	31/576	252/8076	0.001913	0.035874	0.030084	31	All-DEGs
hsa05163	Human cytomegalovirus infection	28/576	225/8076	0.00263	0.046406	0.038916	28	All-DEGs
hsa03010	Ribosome	48/350	158/8076	2.15E-28	5.86E-26	5.49E-26	48	Up-regulation
hsa05016	Huntington disease	35/350	306/8076	1.11E-07	1.51E-05	1.42E-05	35	Up-regulation
hsa05022	Pathways of neurodegeneration - multiple diseases	40/350	475/8076	3.47E-05	0.002842	0.002663	40	Up-regulation
hsa05014	Amyotrophic lateral sclerosis	33/350	364/8076	4.16E-05	0.002842	0.002663	33	Up-regulation

hsa05012	Parkinson disease	25/350	249/8076	7.25E-05	0.003957	0.003708	25	Up-regulation
hsa04714	Thermogenesis	23/350	231/8076	0.00016	0.007271	0.006813	23	Up-regulation
hsa00190	Oxidative phosphorylation	16/350	133/8076	0.000192	0.007485	0.007013	16	Up-regulation
hsa05020	Prion disease	25/350	273/8076	0.000311	0.01062	0.009951	25	Up-regulation
hsa00240	Pyrimidine metabolism	9/350	57/8076	0.000693	0.021017	0.019692	9	Up-regulation
hsa00330	Arginine and proline metabolism	8/350	51/8076	0.001428	0.038988	0.03653	8	Up-regulation
hsa05205	Proteoglycans in cancer	20/226	205/8076	1.03E-06	0.000253	0.0002	20	Down-regulation
hsa04810	Regulation of actin cytoskeleton	20/226	218/8076	2.72E-06	0.000331	0.000263	20	Down-regulation
hsa04919	Thyroid hormone signaling pathway	14/226	121/8076	6.43E-06	0.000523	0.000415	14	Down-regulation
hsa04390	Hippo signaling pathway	14/226	157/8076	0.000122	0.004942	0.003923	14	Down-regulation
hsa04360	Axon guidance	15/226	181/8076	0.000159	0.005548	0.004404	15	Down-regulation
hsa05100	Bacterial invasion of epithelial cells	9/226	77/8076	0.000277	0.008101	0.00643	9	Down-regulation
hsa04141	Protein processing in endoplasmic reticulum	14/226	171/8076	0.000299	0.008101	0.00643	14	Down-regulation
hsa04151	PI3K-Akt signaling pathway	22/226	354/8076	0.000358	0.008729	0.006929	22	Down-regulation
hsa04110	Cell cycle	11/226	124/8076	0.000679	0.015057	0.011952	11	Down-regulation
hsa04730	Long-term depression	7/226	60/8076	0.001339	0.027231	0.021616	7	Down-regulation
hsa04611	Platelet activation	10/226	124/8076	0.002429	0.04529	0.03595	10	Down-regulation
hsa05225	Hepatocellular carcinoma	12/226	168/8076	0.002599	0.04529	0.03595	12	Down-regulation
hsa03010	Ribosome	12/137	158/8076	1.4E-05	0.002992	0.002455	12	PE_related-DEGs
hsa05225	Hepatocellular carcinoma	12/137	168/8076	2.59E-05	0.002992	0.002455	12	PE_related-DEGs
hsa05016	Huntington disease	14/137	306/8076	0.00066	0.040278	0.033038	14	PE_related-DEGs
hsa05205	Proteoglycans in cancer	11/137	205/8076	0.000697	0.040278	0.033038	11	PE_related-DEGs
hsa04151	PI3K-Akt signaling pathway	15/137	354/8076	0.000935	0.043187	0.035423	15	PE_related-DEGs
